# Measuring the effects of the COVID-19 pandemic on Diné and White Mountain Apache school personnel, families, and students: protocol for a prospective longitudinal cohort study

**DOI:** 10.1186/s12889-022-13208-7

**Published:** 2022-08-04

**Authors:** Joshuaa D. Allison-Burbank, Allison Ingalls, Paul Rebman, Rachel Chambers, Renae Begay, Ryan Grass, Alicia Tsosie, Shannon Archuleta, Allison Barlow, Francene Larzelere, Laura Hammitt, Lauren Tingey, Emily Haroz

**Affiliations:** Center for American Indian Health, Department of International Health, Johns Hopkins Bloomberg School of Public Health, 415 N. Washington St., 4th Floor, Baltimore, MD 21231 USA

**Keywords:** COVID-19, American Indian, Schools, In-person learning, Mental health, Educational attitudes, Health and wellness

## Abstract

**Background:**

This paper describes the protocol for a longitudinal cohort study, “Project SafeSchools” (PSS), which focuses on measuring the effects of COVID-19 and the return to in-person learning on Diné (Navajo) and White Mountain Apache (Apache) youth, parents, and educators. The early surges of the COVID-19 pandemic led to the closure of most reservation and border town schools serving Diné and Apache communities. This study aims to: (1) understand the barriers and facilitators to school re-opening and in-person school attendance from the perspective of multiple stakeholders in Diné and Apache communities; and (2) evaluate the educational, social, emotional, physical, and mental health impacts of returning to in-person learning for caregivers and youth ages 4–16 who reside or work on the Diné Nation and the White Mountain Apache Tribal lands.

**Methods:**

We aim to recruit up to *N =* 200 primary caregivers of Diné and Apache youth ages 4–16 and up to *N =* 120 school personnel. In addition, up to *n =* 120 of these primary caregivers and their children, ages 11–16, will be selected to participate in qualitative interviews to learn more about the effects of the pandemic on their health and wellbeing. Data from caregiver and school personnel participants will be collected in three waves via self-report surveys that measure COVID-19 related behaviors and attitudes, mental health, educational attitudes, and cultural practices and beliefs for both themselves and their child (caregiver participants only). We hypothesize that an individual’s engagement with a variety of cultural activities during school closures and as school re-opened will have a protective effect on adult and youth mental health as they return to in-person learning.

**Discussion:**

The results of this study will inform the development or implementation of preventative interventions that may help Diné and Apache youth and their families recover from the negative impact of the COVID-19 pandemic, and positively impact their health and wellness.

**Supplementary Information:**

The online version contains supplementary material available at 10.1186/s12889-022-13208-7.

## Background

American Indians/Alaska Natives (AI/ANs) experienced the highest rates of COVID-19 disease and death in the United States (US) during the early surges of the COVID-19 global pandemic [1]. AI/ANs have experienced more severe COVID-19 illness, with higher death rates than the general US population [6, 52]. Despite these trends, AI/AN communities have shown remarkable resilience. Many AI/AN communities experienced high vaccine uptake in 2021 compared to other racial and ethnic minority groups, an accomplishment that owes itself to a deep reservoir of strength and collective effort [15].

However, the COVID-19 pandemic also presents unique mental and psychosocial health challenges for AI/AN communities, exacerbating existing mental health disparities. The catastrophic history of infectious diseases, including those intentionally introduced as an act of settler colonialism, underlies historical trauma that continues to impact AI/AN people today. In some AI/AN communities, historical trauma is defined as the intergenerational psychological and biological response to the loss of Indigenous lands, peoples, languages, and cultures due to settler colonization and genocide [5, 41]. Several studies have found that historical trauma has negative intergenerational effects, including an increased likelihood of depressive symptoms, school delinquency, suicidal thoughts and behaviors, and substance use among AI/AN youth [9, 10, 30, 42, 47].

While the COVID-19 pandemic is global in its instillation of fear and anxiety, within the context of historical trauma, the physical and psychological threat of infectious diseases on individuals, families, communities, and whole Tribal Nations is particularly acute for AI/AN communities. The collective memories of past and current trauma during an ongoing pandemic have the potential to further intensify existing mental health and educational disparities across Tribal Nations. This confluence of trauma requires a unique understanding and response from public health professionals and educators working to help AI/AN communities to cope with and heal from pandemic-related stressors.

For families and youth across the US, school closures related to COVID-19 have increased general pandemic-related stress. At the beginning of the COVID-19 pandemic, vast numbers of elementary, middle, and high schools closed for in-person learning. School closures left millions of children and adolescents without the structure, support, resources, caring adults, and role models critical to healthy growth and development. Most students in the US started the 2020-2021 academic year virtually and remained in some type of virtual instruction setting throughout the year (U.S. Census Bureau, 2020) [31]. Moreover, enrollment in schools decreased with little understanding of what happened to those children and adolescents who were no longer enrolled [25]. For example, across the US, public school enrollment dropped by 3%, representing an estimated 1.5 million students who did not show up to schools as expected.

School closures not only resulted in decreased enrollment but are predicted to have massive ripple effects for decades to come, including missed learning opportunities due to chronic absenteeism during remote learning and continued learning difficulties due to pandemic-related stressors [13]. Collectively, these COVID-19-related school closures will have long-lasting effects, with primary school closures estimated to result in 13.8 million years of lost life [7] and an estimated $2.5 trillion reduction in future earnings [36]. On average, students are estimated to have started the 2020–2021 school year with approximately 32% lower learning gains in reading and 50–63% lower learning gains in math, relative to pre-pandemic years [27]. As a result of interrupted in-person learning, AI/AN students are experiencing exceptional losses in reading and math achievement including poor reading proficiency in elementary students and limited school readiness skills for students starting kindergarten during the pandemic [37]. School closures did not just impact students. Parents have been greatly impacted by the stress of having to miss work to help children with school or try to work simultaneously with virtual students at home, uncertain job security, and increased financial burden, among other challenges unique to parents of school-aged children ([45]; National Board for Professional Teaching Standards, n.d.). Further, school closures have been found to be associated with worse mental health among racially and ethnic minority populations [19].

With regards to the Navajo Nation (Diné) and the White Mountain Apache Tribe (WMAT), two Tribal Nations in the Southwest US, the COVID-19 pandemic resulted in all schools on each reservation being closed for in-person learning for most students from March 2020 through the end of the 2019–2020 academic year; however, due to poor broadband connectivity in many locations and a lack of computer equipment for children at home, virtual learning was not possible at times for many children. Most of these schools remained in a virtual learning format at the beginning of the 2020–2021 academic year. Both within and outside of AI/AN communities, it is well-documented that school attendance and attachment among youth have a profound protective impact on their physical and mental health [26, 34]. In the White Mountain Apache community specifically, pre-pandemic research conducted by members of this study team has shown that school attachment is protective and can reduce youth binge substance use, marijuana use, suicide ideation, and suicide attempt [44].

Reopening schools is paramount to ensuring the safety and well-being of children and adolescents, especially for AI/AN and other racial/ethnic minority students who benefit greatly from school-based resources for psychosocial support and socialization. For example, there was significant concern over increases in suicidal behaviors among Diné and White Mountain Apache (Apache) youth during the pandemic, a pattern that has been corroborated at the national level [49]. In addition, suicide risk is predicted to be further exacerbated by stressed linked to the COVID-19 pandemic. On the Diné Nation, students have reported increased anxiety about attending school in-person during the COVID-19 pandemic [35]. Simultaneously, AI/AN school-age children are more likely to experience learning difficulties and receive special education services and disruption in learning can further increase this risk. This learning risk in addition to decreased learning time has increased social emotional and behavioral stress for culturally and linguistically diverse students [13], particularly for AI/AN youth. Moreover, the disproportionate burden of COVID-19 morbidity and mortality has resulted in a significant and substantial grief burden as youth and families return to in-person learning. This includes loss of primary caregivers, which AI/AN youth have experienced 4.5 times more than non-Hispanic White youth [6]. Navigating lost learning, the ongoing uncertainties of the pandemic, and the burden of grief, while simultaneously managing layered public health precautions, such as masking, social distancing, and testing, will inevitably have a substantial negative impact on schools, personnel, families, and students. The purpose of this paper is to describe the protocol for a longitudinal cohort study, called Project Safe Schools (PSS), which focuses on measuring the effects of the COVID-19 pandemic and the return to in-person learning on Diné and Apache youth, parents, and educators.

## Study aims

This study aims to: (1) understand the barriers and facilitators to school re-opening and in-person school attendance from the perspective of multiple stakeholders in the Diné and Apache communities; and (2) evaluate the educational, social, emotional, physical, and mental health impacts of returning to in-person learning for caregivers and youth ages 4–16 years who reside or work on the Diné Nation and White Mountain Apache tribal lands. We will utilize participatory approaches, including having all aspects of the study design, implementation, and evaluation guided by Community Advisory Boards (CABs) and working with trained public health researchers from the participating communities. This prospective longitudinal cohort study will aim to recruit up to *N =* 200 Diné and Apache families and up to *N* = 120 school personnel and follow them for up to 2 years as they return to in-person learning and continue to navigate the uncertainty of the ongoing COVID-19 pandemic. We hypothesize that an individual’s engagement with cultural activities will have a protective effect on caregiver and youth mental health as they return to in-person learning. Consistent with a public health approach, the results of this study will inform the development and implementation of preventive interventions that may help AI/AN youth and their families recover from the negative impact of the COVID-19 pandemic and positively impact their health and wellness. In addition to these aims, this study will continue to strengthen the public health infrastructure and build long-term collaborative partnerships with schools that serve Diné and White Mountain Apache Nations.

## Methods/design

We will use *The Strengthening the Reporting of Observational Studies in Epidemiology (STROBE) Cohort Studies Checklist* to present the methods of this study protocol [48]. See Additional file 1 for the completed checklist.

### Partner communities and study setting

#### The Diné Nation & White Mountain Apache Tribe

These two federally recognized tribes are in the southwestern US. The White Mountain Apache Tribe is in present-day eastern Arizona on the Fort Apache Indian Reservation. The Diné Nation is the largest federally recognized tribe and extends across Arizona, New Mexico, and Utah. Both Tribal Nations experience significant socioeconomic, educational, physical and mental health disparities compared to US All Races. Despite these disparities, the Diné and WMAT are resilient people who maintain their traditional language and ways of life and prioritize youth wellness and continued resilience within their communities. Many schools on the Diné Nation and WMA Tribal lands now include instruction in the Diné and Apache languages and embed cultural teachings into daily learning. This project focused on the Diné communities of Shiprock, NM, Chinle, AZ, and Tuba City, AZ and the entire White Mountain Apache Tribal lands.

### Study context

This study is nested within a larger study focused on understanding the implementation of COVID-19 testing in schools as an added mitigation strategy to identify COVID-19 cases. Funding from the National Institutes of Health Rapid Acceleration of Diagnostics (RADx®) Programs (Grant #1OT2HD107543) allowed for support to schools for the implementation of COVID-19 testing and research related to returning to in-person learning.

### Community engaged research process

This study will utilize a community-based participatory research approach to maximize opportunities for community involvement in the study design and implementation [8]. Prior to the launch of the study, local approvals were sought and obtained. On the Diné Nation, this included obtaining input from our Community Advisory Boards, obtaining local approvals from community leaders (e.g., Chapter Houses, Regional Agency Councils), school districts and the Navajo Nation Human Research Review Board. For WMAT, local approvals were obtained from the Tribal Health Advisory Board and Tribal Council. To enhance collaboration with the health system, additional support and permission were obtained from local Indian Health Service Units and Tribal Health Organizations. Additional approval was received from the Johns Hopkins Bloomberg School of Public Health Institutional Review Board (IRB No: 14911/MOD 1874, Approval Date: December 21, 2021). All project activities are guided by a Community Advisory Board for each participating Tribal Nation. These CABs consist of community members, medical professionals, and educators from each of the communities. The CABs met prior to the launch of the study to advise on study methods, priority outcome domains, and culturally responsive community engagement. The CABs will be engaged throughout the study process to be informed of progress, preliminary findings and to provide guidance on how to interpret findings and further areas to enhance the research.

### Study population

Study participants will be primary caregivers (age 18+; up to *N =* 250) of youth ages 4–16 years recruited through convenience sampling in partnership with schools and community-based efforts. Minors (ages 11–16; up to *N* = 120) and a subset of primary caregivers (up to *N =* 120) will be recruited from this pool of caregivers to participate in qualitative interviews. In addition, we will recruit up to *N =* 120 school personnel (e.g., teachers, staff, administrators; ages 18+) to participate in qualitative interviews and surveys at two time points.

#### Inclusion and exclusion criteria

Inclusion criteria by participant type are included in Table 1. Individuals who are unable to provide consent due to cognitive or language deficits will be excluded from this study.

**Table 1:** Project SafeSchools cohort study inclusion criteria, by participant type

Participant Type	Inclusion Criteria
Parents/Primary Caregivers Complete survey and qualitative interview	• A parent or primary caregiver of a child aged 4–16 years who is eligible to attend a school that serves Diné or Apache nation members. • 18 years old or older • Consent to all study activities
School Personnel Complete qualitative interview	• An employee of a school that serves Diné or Apache nation members. • 18 years old or older • Consent to all study activities
Minors Complete qualitative interview	• 11–16 years old • Enrolled in a school that serves Diné or Apache nation members • Parental consent to participate in study activities • Assent to participant in all study activities

#### Recruitment

Participants for this study will be recruited by local trained Diné and Apache research personnel through existing partnerships with participating schools, attending local events, through social media, and connections to other service agencies and groups on each reservation.

### Data collection

Parent/primary caregivers and school personnel enrolled and orally consented into the study will participate in both quantitative and qualitative data collection. Minors enrolled in the study will be assented and will only participate in qualitative data collection. Quantitative data collection will take place at three time points, with approximately 6 months between the first two timepoints, and 9–12 months between the second and last time point. School personnel will only participate in the first two time points. Qualitative data collection will take place following the first two time points.

Quantitative data collection will focus on participant demographics, school enrollment, COVID-19 related behaviors and attitudes, cultural practices, mental and behavioral health, and education perceptions. School personnel will self-report information on a subset of domains. Parents/primary caregivers will provide self-report data and report on one child under their care. Domains covered for each participant and the relevant measures are included in Table 2.

**Table 2:** Project SafeSchools cohort study quantitative assessment domains and measures

Domain	Measures	Time Points		
		B	W2	W3^a^
**Parent/Primary Caregiver and School Personnel Self-Report**				
Age, gender, household composition, employment, etc.	Internally developed questionnaire	X		
Current school enrollment and promotion information	Internally developed questionnaire	X	X	X
COVID-19 related attitudes and behaviors on COVID-19 testing, safety, and vaccinations	Internally developed questionnaire	X	X	X
Acceptability, Appropriateness, Feasibility of COVID-19 Testing	Acceptability of Intervention Measure (AIM), Intervention Appropriateness Measure (IAM), and Feasibility of Intervention Measure (FIM) [51]	X	X	X
Attitudes towards in-person and virtual learning	Internally developed questionnaire	X	X	X
Food Insecurity	Household food security tool [4]	X		X
Access to Services	Internally developed questionnaire	X	X	X
Alcohol and Tobacco	Internally developed questionnaire	X	X	X
Adult distress	Kessler 6 [24]	X	X	X
**Parent/Primary Caregiver Self-Report**				
Posttraumatic stress	PTSD Checklist for DSM-5 (PCL-5) [3]	X	X	X
Anxiety	PROMIS Anxiety Short Form [38]	X	X	X
Depression	Center for Epidemiologic Studies Depression Scale Revised – 10 items (CESDR-10) [16]	X	X	X
Self-esteem	Rosenberg Self-esteem Measure [39]	X	X	X
Cultural identity	Leach’s in-group identification scale [28]	X	X	X
Adverse Childhood Experiences	Native American adapted version of ACEs questionnaire [11]	X	X	X
Suicidal thinking/behavior	Adapted questionnaire based on youth risk behavior surveillance system (YRBSS) [23]	X	X	X
Communal mastery	Multicultural Mastery Scale [12]	X	X	X
Hope	The Trait Hope Scale [43]	X	X	X
Culture and home routines	Internally developed questionnaire	X	X	X
**Parent/Primary Caregiver Report on Child**				
Mental and behavioral health of child	Strengths and difficulties questionnaire (SDQ) [14]	X	X	X
Posttraumatic stress	Child and Adolescent Trauma Screen (CATS) [40]	X	X	X
Anxiety	Screen for Child Anxiety Related Emotional Disorders (SCARED) [2]	X	X	X
Suicidal thinking/behavior	Adapted questionnaire based on youth risk behavior surveillance system (YRBSS) [23]	X	X	X
Resilience	Child & Youth resilience Measure-Revised Person Most Knowledgeable version [22]	X	X	X
Self-efficacy in schools	Internally developed and combined with unpublished research from [46]	X	X	X
Educational and learning attitudes	Adapted Perception of Barriers to education [32]	X	X	X

^a^Wave 3 will only include parents/primary caregivers

All quantitative data collection and qualitative data tracking for both parents/caregivers and school personnel will be collected and managed using REDCap electronic data capture tools [17, 18]. For participants who score above pre-established cutoffs on mental health instruments or indicate suicide risk, an additional email alert to research staff will occur, and prompt follow-up will take place according to an established mental health response plan.

#### Parents/primary caregivers

Parents and primary caregivers (~ *N =* 250) will complete a quantitative survey administered at study enrollment and at two additional time points (i.e., Waves 2 and 3, also see Table 2). Surveys may be administered by email through a secure link, in-person, or completed independently via tablet, or on paper. A sub-sample of parents/caregivers of children 11–16 years old, up to 30 at each site, will be asked to participate in two qualitative interviews. Qualitative interviews will take place at study enrollment and approximately 6 months after enrollment and will explore participant’s experiences and perspectives on school closures, virtual learning and the return to in-person learning.

#### School personnel

School personnel (*N =* 120) will complete two quantitative surveys administered at study enrollment and approximately 6 months post-enrollment (see Table 2). A sub-sample of school personnel, up to 30 at each site, will be asked to participate in two qualitative interviews. Qualitative interviews will focus on returning to in-person instruction and COVID-19 testing and vaccine uptake, components of the larger study.

#### Minors

Minor participants (ages 11–16; up to *N =* 120), whose parent or primary caregiver is enrolled in the study, will be selected for two in-depth qualitative interviews. Minor participants will complete one interview after parental/caregiver enrollment and one ~ 6 months later. These semi-structured interviews will focus on student perspectives on returning to school and COVID-19 testing.

#### Participant safety monitoring and referrals

Due to the sensitive nature of many of the assessment measures in this study, study staff will follow specific procedures for responding to self-reported participant safety concerns. In addition, all study staff are required to participate in suicide prevention training to ensure participant safety. Community site leads completed Applied Suicide Intervention Skills Training (ASIST), which provides evidence-based support to help intervene with individuals at high-risk for suicide [29].

At the end of each study visit where quantitative data collection is completed, a notification will appear to the study staff member if the participant’s answers indicate risk for severe distress and/or risk of suicidal behaviors. In addition, for primary caregivers who are reporting on an index child, similar alerts will be triggered if they report any high-risk mental health challenges for that child. If suicide risk is endorsed, study staff will respond in a graduated fashion based on the approved, site-specific safety protocol. In most cases, this means immediately contacting a supervisor who will review the scores and other potentially relevant information (i.e., recency and severity of behavior reported, family support, whether they have an appointment/have seen anyone for counseling, comments they may have made during visit, etc.) and provide assisted referrals to appropriate local mental and behavioral health services. If the participant is in immediate danger, emergency services will be called.

Alerts for non-suicide related mental health concerns include adult participants responding with scores above validated cutoffs on the Kessler-6 (school personnel & parent/caregiver participants), CESD-10-R, PCL, or PROMIS anxiety measure (parent/caregiver participants). Similar alerts are also sent if adult participants indicate their child (parent/caregiver participants) meets the standard cutoffs on the SDQ, CATs, or SCARED. Participants who have these alerts will receive a referral to local mental health services and be assisted with contacting these services. As part of our study protocol, we will track all mental health and suicide risk related alerts, including what was done during follow-up procedures.

### Analysis plan

#### Qualitative analysis

We are using the Theoretical Domains Framework (TDF) of behavior change [33] to understand what drives a parent/caregiver, teacher/staff, or youth to return to in-person learning and/or participate in school-based testing. Qualitative interviews will be audio-recorded, transcribed using a standard transcription service (e.g., Rev.com), then thematically coded using a priori coding (guided by the TDF) and emergent coding generated during the analysis process. Data will be synthesized across type of participant (e.g., parent/caregiver, school employee) to explore similarities and differences by stakeholder group. The first 10 interview transcripts will be double coded by two research assistants trained by an expert in qualitative research who will check the coding for inter-rater reliability. After coding these transcripts, the interviewers will compare their codes for consistency and then resolve discrepancies through consensus. This will also inform revisions to the codebook as needed. The coders will then independently code the remaining transcripts. Data will be summarized using tables and thematic descriptions and saturation will be determined when no new themes emerge.

#### Quantitative analysis

Data for quantitative analysis will come from parent/caregiver and school personnel surveys. We will use Mixed-effects Regression Models (MRMs) to account for repeated measurements from the same participant over time and site-level clustering. Planned analyses include exploring time-stable and time-varying factors that may influence adult and youth mental health outcomes. All models will control for basic demographic variables, such as age and gender. Additional analyses will also occur depending on guidance from our CABs.

Every effort will be made to generate complete data; however, inevitably some data will be missing, and the methods recommended by Schafer and Graham (2002) will be used to evaluate missing data assumptions and guide the analyses. Based on the amount of missing data and evidence for a Missing At Random (MAR) versus Missing Not At Random (MNAR) mechanisms, missingness will be addressed through multilevel multiple imputation or inverse probability weighting, as appropriate. The missing data approach will be finalized before the outcome analysis.

#### Statistical power

Given the multilevel nature of our data (i.e., timepoints, nested within persons, nested within sites) statistical power was calculated utilizing techniques recommended by Hox et al. [21] and Hedges and Rhoads [20]. This process has three steps. For Step 1, we estimate power for a single level regression model. This serves as the *target* sample size. In our case, using G*Power, to detect a small-medium effect for a single-level regression (f^2^ = 0.035), our type I error rate (alpha; *a*) set to *a =* 0.05, power set to 80%, and two predictors, we calculated a *target* sample size of *n* = 279. For Step 2, we compute the *actual* sample size for the proposed study. With *n =* 279 participants and 3 measurement timepoints, there are 837 (non-independent) observations. Finally, in Step 3, we penalize the actual sample size for the nesting effect using the design effect formula (i.e., n_eff_ = n / [1 + {n_clus_ – 1}ρ]). This provides the *effective* sample size. If the *effective* sample size is greater than or equal to the *target* sample size calculated in Step 1, then there is sufficient power to detect the effect of interest. To calculate the *effective* sample size, we used a conservative estimate for ρ (0.5), which resulted in our *actual* sample size (step 2) providing the statistical power of the equivalent of *n =* 417 independent observations as our *effective* sample size which is substantially larger than the *target* sample size indicating adequate power to detect at least an effect of f^2^ = 0.035 for a given association.

Given challenges with study recruitment during a pandemic, we further explored what the minimum sample size would be to detect at least an effect of f^2^ = 0.035 using the approach described above. We then adjusted this minimal necessary sample size to account for 30% attrition over the course of the study. Our final minimal sample size needed is at least *N =* 266 participants at baseline to be able to detect an effective size of f^2^ = 0.035 on a continuous outcome using multilevel modeling.

## Discussion

Project SafeSchools aims to measure the impact of the Covid-19 pandemic on Diné and Apache family and youth and school personnel well-being as students return to in-person learning. The global pandemic has greatly impacted AI/AN communities by closing schools and forcing school-age children to learn in unconventional settings. This included further complications associated with limited access to broadband Internet and electronic equipment (e.g. laptops, desktops, etc.) essential for successful remote learning. Continued uncertainty due to ongoing circulation of SARS-CoV-2 variants presents unique challenges as schools serving reservation communities attempt to safely re-open and meet the educational needs of students. The mental health burden will likely be an added consideration for school districts in the upcoming years requiring a multidisciplinary public health response to meet the needs of Diné and Apache students and families. Project SafeSchools will utilize psychiatric epidemiology and community-based participatory research to assist with COVID-19 pandemic response and recovery efforts across AI/AN communities. Furthermore, this study seeks to identify facilitators of youth health and wellness and community resilience. It is predicted that cultural routines, such as participation in traditional activities, farming, and speaking heritage languages, will act as buffers to enduring periods of elevated stress linked to the COVID-19 pandemic. This study will foster continued collaboration among educators, public health researchers, and Tribal leaders on child and adolescent mental health and school-based supports.

### Limitations

Although we believe this study has important implications for understanding the impacts of COVID-19 stress and school closures on adult and youth mental health in AI/AN communities, there are several limitations. First, the study utilizes convenience sampling to identify participants. While convenience sampling was deemed most feasible, we continuously examine the characteristics of our study sample and aim to prioritize demographic subgroups that are underrepresented (e.g., male caregivers, virtual learners, etc.). Further, this study utilizes parental reports for youth mental health outcomes. Parental reports and youth self-reports may differ for youth mental health outcomes and may measure different aspects of problems or constructs (43). Finally, while most of our measurement instruments were selected based on previous use of these tools in AI/AN populations [50], not all our tools, nor their clinical cut-off points have been validated in Diné and Apache samples. We will analyze the psychometric properties as part of the baseline analysis and use that information to adjust instruments as needed for waves 1 and 2.

This study will be one of the first cohort studies in AI/AN communities aimed at measuring the effects of the COVID-19 pandemic and return to in-person learning on youth, caregivers, and educators. Results of this study will be used to further advise educators, health care providers, and Tribal leaders on approaches to promoting youth wellness during the upcoming years of recovery from the COVID-19 pandemic.

## Supplementary Information

**Additional file 1.**

## Data Availability

Not applicable.

## Abbreviations

AI/AN: American Indian/Alaska Native
Apache: White Mountain Apache
ASIST: Applied Suicide Intervention Skills Training
CAB: Community Advisory Board
CATS: Child and Adolescent Trauma Screen
CESDR-10: Center for Epidemiologic Studies Depression Scale Revised
MAR: Missing at Random
MNAR: Missing Not at Random
MRM: Mixed-methods Regression Models
PCL: PTSD Checklist
PROMIS: Patient-Reported Outcomes Measurement Information System
RADx®: Rapid Acceleration of Diagnostics initiative
REDCap: Research Electronic Data Capture
SCARED: Screen for Child Anxiety Related Disorders
SDQ: Strengths and Difficulties Questionnaire
STROBE: Strengthening the Reporting of Observational Studies in Epidemiology
TDF: Theoretical Domains Framework
US: United States
WMAT: White Mountain Apache Tribe
